# Alcohol Dependence Syndrome With Bipolar Affective Disorder and Hypomanic Current Episode: A Case Report

**DOI:** 10.7759/cureus.55994

**Published:** 2024-03-11

**Authors:** Sharayu P Wankhade, Joel Gibbs

**Affiliations:** 1 Psychology, Jawaharlal Nehru Medical College, Datta Meghe Institute of Higher Education and Research, Wardha, IND

**Keywords:** pharmacotherapy, psychotherapy, hypomania, bipolar affective disorder (bpad), alcohol dependence syndrome (ads)

## Abstract

Studies have revealed that individuals with bipolar I and bipolar II have a past of substance abuse. The co-occurrence of bipolar disorder and alcoholism is frequent. Although various arguments have been put forward to explain the relationship between these disorders, it is still not fully understood. Since substance abuse is prevalent among bipolar patients, it would be beneficial to investigate the impact of substance abuse on clinical characteristics, as well as the progression of the illness. Thus, this study was carried out to investigate a case of alcohol dependence with bipolar disorder. A 49-year-old male visited the psychiatry outpatient department and then was admitted. The patient's chief complaints were alcohol consumption, cigarette smoking, daily drinking for 35 years, irritability/aggressiveness, boastful talk, overspending, and decreased need for sleep from the last 20 days. According to the literature, self-medicating with alcohol is not an effective treatment for alcoholism, unless it is being used to alleviate the psychological and neurochemical effects caused by alcohol. However, there has been limited research on how to treat individuals who have both alcoholism and another medical condition. A few studies have looked at the impact of medications like valproate, lithium, and naltrexone, as well as psychosocial interventions, in treating patients with bipolar disorder and alcoholism. However, more research is necessary to fully understand the best approach.

## Introduction

More research is needed on how substance abuse affects the progression of bipolar disorder, especially since many bipolar patients also struggle with substance abuse. Differentiating between bipolar disorder before and after substance abuse begins is important in understanding the overall course of the illness [1]. Bipolar disorder causes extreme mood swings from euphoria to severe depression and affects 1-2% of the population. It often goes undiagnosed and untreated for long periods, with some patients waiting up to 10 years to receive treatment [2]. Alcoholism is a strong desire for alcohol, leading to physical dependence and loss of control. Around 14% of the general population experience it. Alcohol abuse neglects responsibilities, occurs in dangerous situations, and causes legal and relationship problems. It often leads to alcohol dependence in early adulthood [2]. Bipolar disorder causes extreme mood swings from euphoria to severe depression and affects 1-2% of the population. It often goes undiagnosed and untreated for long periods, with some patients waiting up to 10 years to receive treatment [2]. Alcohol dependency increases the risk of bipolar disorder by 3% compared to the general population's 1%. This co-occurrence is surpassed by the occurrence of antisocial personality disorder (ASPD) in alcohol dependence [3]. It was categorized as alcohol use disorder in the Diagnostic and Statistical Manual (DSM 5) combining alcohol diagnosis of abuse and dependence under one study. In the manic phase of bipolar disorder, individuals may experience marital problems and feel a sense of urgency to resolve them [4-6].

According to a study, valproate therapy can reduce heavy drinking in patients who have both bipolar disorder and alcohol dependence. This suggests that valproate, an anticonvulsant mood stabilizer, could have practical use in treating bipolar disorder and alcohol dependence simultaneously [7]. Psychotherapeutic and psychosocial interventions are effective in treating substance use disorders. Cognitive behavioral therapy and contingency management have shown success. These principles can also be used to treat affective disorders [8]. This article reviews clinical studies on conventional mood stabilizers' effectiveness in treating alcohol withdrawal, relapse prevention, and bipolar disorder with comorbid alcoholism [9]. A French national multisite collaborative study on the clinical epidemiology of Mania (EPIMAN) in bipolar I disorder examined various aspects of the condition and aimed to evaluate prevalence rates of alcohol use disorder, alcohol use disorder - bipolar disorder, and bipolar disorder - alcohol use disorder in a large sample of bipolar I patients [10,11]. This study compared bipolar patients with and without alcohol use disorder (AUD) and identified risk factors for the onset and co-occurrence of bipolar disorder and AUD, focusing on temperamental components [12]. 

## Case presentation

Patient information

A 49-year-old widowed male, educated till graduation, unemployed, belonging to middle socio-economic status, extended family, resident of Hinghanghat was accompanied by his cousin. The presenting complaints were alcohol consumption, cigarette smoking, daily drinking for 35 years, irritability/aggressiveness, boastful talks, overspending, and decreased need for sleep from the last 20 days. The precipitating factor is the death of his wife, the predisposing factor is the history of psychiatric illness in the maternal aunt, and the perpetuating factor of illness in the patient is non-compliance. There was past history of multiple visits and admissions with psychiatrists. His last admission was at Acharya Vinoba Bhave Rural Hospital. 

Personal history

Regarding birth and early development (history of abuse), no reliable informant was available. There was no history of lying, bullying, or trauma in school. The patient has worked in multiple jobs as per the occupational history. He worked in Japan, as a truck driver and exporter of goods, in a bookstore, and taught meditation. The patient got married in 2001 and his wife passed away in 2011 due to breast cancer as per the sex and marital history. No reliable informant was available regarding personality before illness.

Clinical findings

Physical examination revealed blood pressure: 130/80, pulse rate: 108, height: 174 cm, weight: 90kg, and body mass index: 29.7 (overweight). Psychiatric examination revealed that the patient was cooperative, conscious, oriented to time, place, and person, sitting comfortably in bed, inappropriately dressed, unkempt, eye contact initiated and maintained, rapport was established, and speech was average in rate of tone, volume, coherence, and relevance. The patient said that his mood was euthymic but affect was “irritable”. There were grandiose ideas of thought and prolixity of speech, and he denied suicidal ideation and abnormal perception. Higher mental function revealed attention and concentration were aroused but not sustained, there was intact memory, proverb was intact, and there was impaired personal and social judgment, insight being grade 2/5.

Diagnostic assessment

Diagnosis is alcohol dependence syndrome with bipolar affective disorder, and the current episode is hypomanic without psychotic symptoms. Classically, the prognosis in mood disorders is generally described as better than in schizophrenia. The following is a list of positive and negative prognostic variables under mood disorder. Good prognostic factors are abrupt or acute onset, severe depression, typical clinical features, well-adjusted premorbid personality, and good response to treatment. Poor prognostic factors are double depression, co-morbid physical disease, personality disorders or alcohol dependence, chronic ongoing stress, poor drug compliance, and marked mood incongruent features.

Therapeutic intervention

The patient is currently on the following medications, T. Sodium Valproate 750 mg (0-0-1 )HS, T. Aripiprazole 10 mg (0-0-1), T. Lorazepam 2 mg (1-1-2 )TDS, and T. Thiamine 100 mg (2-2-2 )TDS. The psychological treatment plan was to give cognitive behavior therapy (CBT), interpersonal therapy, group therapy, family therapy, and psychosocial rehabilitation.

## Discussion

Additionally, psychosocial therapy can be beneficial in managing mood disorders [4]. It is often used as a supplement to physical therapy and is particularly useful in cases of mild to moderate depression. Cognitive behavior therapy aims to replace negative thoughts and behaviors associated with depression, such as feelings of despair, worthlessness, helplessness, and pessimism, with more positive alternatives [4,5]. Cognitive behavior therapy can be used to treat mild to moderate non-bipolar depression, with or without somatic therapy. Interpersonal therapy aims to pinpoint and explore issues related to relationships, role conflicts, life changes, loneliness, or difficulties with social skills that may contribute to depression. It can be utilized as a standalone treatment or in conjunction with antidepressants to address mild to moderate unipolar depression [4,5]. Psychoanalytic psychotherapy is a form of short-term psychodynamic psychotherapy that aims to change a person's personality rather than just treating their symptoms. While its effectiveness during manic or depressed states is up for debate, this approach can be useful for certain individuals seeking treatment [4,5]. Behavior therapy includes various techniques such as social skills training, problem-solving strategies, assertiveness training, self-control treatment, activity scheduling, and decision-making techniques. It can be helpful in cases of mild depression and may also be used alongside antidepressants for moderate depression [4,5]. For individuals experiencing moderate forms of depression, group therapy could be a helpful option. Group psychotherapy is an effective treatment that provides psychological support and education for those with recurring depressive illness or bipolar disorder [4,5]. When it comes to mood disorders, family therapy has not proven to be effective in treating them. However, it can still be beneficial in educating the family about the illness and the effectiveness of somatic treatment [4,5]. These therapies, on the other hand, can assist in reducing interfamilial and interpersonal challenges. It helps eliminate or adjust stressors, which may aid a faster and more complete recovery.

Bipolar disorder and substance abuse often occur together, possibly due to confusion during diagnosis. Symptoms overlap, leading to misdiagnoses [8]. Alcohol abuse or dependence may alter the presentation of bipolar disorder, resulting in higher rates of certain symptoms such as mixed or dysphoric mania, rapid cycling, and impulsivity. Researchers are still exploring the relationship between the two conditions [9]. Both groups showed similar episode severity in global clinician and self-ratings. Retesting showed high reliability for global self-ratings in both groups. Unipolar depressed patients had high retest reliability, while bipolar patients had more varied responses indicating mood fluctuations [10]. We need prospective validation, which we plan to achieve through the completion of our study's prospective part [11].

Bipolarity is reinforced by several factors, including pre-existing traits, family history of bipolar disorder, and sub-threshold bipolar symptoms. Anticonvulsants can manage acute episodes and address the underlying temperament that drives the desire for mood enhancement and activation. They can effectively mitigate the inclination toward seeking out stimulants and associated behaviors [12]. Patients with affective psychosis face challenges in recovery, including noncompliance with treatment, substance abuse, low socioeconomic status, and poor premorbid function. Positive outcomes within the first year after hospitalization are rare [13]. To better treat bipolar disorder, it's important to identify and intervene early on with sub-syndrome illness, depression, and functional recovery. A combination of medication and psychosocial/rehabilitative interventions applied consistently can improve outcomes [14]. Studies have found few demographic, functional, or treatment factors related to clinical outcomes. Comorbidities, such as substance use disorder and anxiety disorder, have not been studied separately [15]. Clinical settings for mental health and addiction have higher odds of this comorbidity. In prisons, the comorbidity of addiction and severe mental disorders is especially high with disorders like antisocial personality, schizophrenia, and bipolar disorders [16]. Substance abuse is common in bipolar disorder patients and can worsen their illness. Early recognition and treatment can result in better outcomes [17].

Bipolar disorder often co-occurs with anxiety, substance abuse, and eating disorders. Other mental health conditions can make bipolar illness more severe and develop earlier. Research continues to explore the effects of these comorbidities on prognosis and treatment outcomes [18]. Alcohol use may have been a coping mechanism for stress and anxiety in the alcohol use disorder - bipolar disorder group, while stimulant use may have triggered mania in the bipolar disorder - alcohol use disorder group [19]. Alcohol abuse can cause symptoms like depression, anxiety, and antisocial behavior that can resemble genuine psychiatric illnesses. These conditions typically go away after several days or weeks of abstinence. It's important for clinicians to accurately diagnose alcohol-induced psychiatric disorders and rule out independent disorders [20]. When a person suffers from both alcoholism and psychiatric disorders, they may find it challenging to stay sober, may have suicidal tendencies, and may require mental health assistance. As a result, it is crucial to conduct a comprehensive assessment of psychiatric symptoms in alcoholics to mitigate the severity of their condition [20]. Ongoing research is looking into the impact of medications and psychiatric comorbidities [21,22]. As an example, a recent meta-analysis revealed that lithium treatment has minimal negative effects on cognition, which came as a surprise [23]. Exercise caution when categorizing cognitive effect sizes as small, moderate, or large. Magnitudes may not indicate clinical relevance, but rather statistical significance. A cut-off score of 2 standard deviations has been shown to effectively identify significantly impaired individuals [24].

## Conclusions

Alcohol may have been used in the alcohol use disorder - bipolar disorder group to ease the strain and stress brought on by an irritable temperament, anxiousness, and organic problems, which resulted in the initial depressive episode. Alcohol drinking was a consequence of the intensity of mania in the bipolar disorder - alcohol use disorder group and stimulant use may have been the cause of the initial manic episode. There's a chance that the routes that lead to alcohol use disorder - bipolar disorder or bipolar disorder - alcohol use disorder are different. Anxiety and organic problems can be linked to irritable temperament as a source of conflicts with the environment in alcohol use disorder - bipolar disorder, which can result in alcohol and sedative usage as a coping mechanism for tension and stress. Comorbid substance use disorder and particularly alcohol use disorder are more the norm than the exception in bipolar disorder. Although they are still rare, pharmacological and integrated psychotherapy methods that give equal weight to both illnesses are advised. The finest, but as of yet inadequate, evidence-based psychosocial therapies are cognitive behavior therapy and image-guided therapy. Mood stabilizers like valproate and lithium should continue to be the first line of treatment for borderline depression (bipolar disorder), with supportive medication focused mostly on bipolar disorder. To create trustworthy treatment strategies for comorbid alcohol use disorder and bipolar disorder, further studies are necessary.
